# ON-SITE monitoring OF BVOCS emission in Tremiti island, Italy

**DOI:** 10.1016/j.heliyon.2023.e23822

**Published:** 2023-12-19

**Authors:** Martina Fattobene, Fabrizio Papa, Raffaele Emanuele Russo, Silvia Zamponi, Paolo Conti, Fabio Taffetani, Adelmo Sorci, Fuyong Liu, Mario Berrettoni

**Affiliations:** a School of Science and Technology, Chemistry Division, University of Camerino, Via Madonna delle Carceri – ChIP, Camerino (MC), 62032, Italy; b Dipartimento di Scienze Agrarie, Alimentari e Ambientali, Università Politecnica delle Marche, Via Brecce Bianche, Ancona (AN), 60131, Italy; c Laboratorio del Ma.Re, Via A. Vespucci, Isole Tremiti (FG), 71040, Italy; d Zhengzhou University of Light Industry, Zhengzhou, 45000, China

**Keywords:** BVOCs, Adsorbent materials, Forest therapy, Sea spray aerosol, Dynamic flux chamber

## Abstract

A measurement campaign was conducted on San Domino Island, part of the Tremiti Islands archipelago, located in Foggia, Italy. The area is almost entirely covered by vegetation, dominated by the following main species: *Juniperus turbinata, Helichrysum italicum, Myrtus communis, Rosmarinus officinalis, Pistacia lentiscus and Pinus halepensis*.This study focused on the BVOCs emitted by plants and the ground, employing a simple, economical, and efficient sampling and analysis method. The main known BVOC species emitted by Mediterranean plant species as α-pinene, β-pinene, camphene and limonene were detected. The measurements highlighted a daily complementarity between plant and soil emissions. The daily variations in BVOCs emitted by both plants and the soil are differ, ensuring an almost constant concentration throughout the day. At the same time, the composition of sea spray aerosol (SSA) was also measured.

The measurement sites were selected based on botanical characterization to account for the predominant species on San Domino Island, and the sampling was conducted at human height to accurately identify the species for potential use.

The combination of beneficial effects of the substances emitted by plant species and soil, along with the simultaneous presence of SSA, are factors that could enhance the effectiveness of forest therapy in a previously unexplored location.

## Highlights

•Determination of BVOCs in Tremiti Island.
•Sampling and analysis of BVOCs emitted by plant and soil.
•Monitoring of daily trend emission of BVOCs from soil and vegetation.

## 1 Introduction

Biogenic Volatile Organic Compounds (BVOCs) are natural carbon-containing compounds that are released from the Earth's surface into the atmosphere. This category encompasses a wide range of organic species emitted by vegetation, soils, and oceans [1]. Plants emit BVOCs, such as terpenes, monoterpenes (MTs), and sesquiterpenes (SQTs) from their foliage for a variety of reasons. These include intra- and inter-plant communication, attraction of pollinators and seed dispersal animals, and protection against biotic and abiotic stress [2,3].

The emission of biogenic VOCs is greatly affected by environmental conditions such as light, temperature [4], humidity, and seasonality. It is highly sensitive to climate change and environmental stress. The extent of this influence varies based on the compound category. Isoprene emission, for example, is strongly dependent on photosynthetically active radiation “PAR”, which is the portion of the spectrum (400–700 nm) that triggers photosynthesis [5].

The response of isoprene to PAR is hyperbolic, indicating that emissions increase with rising light intensity until they reach a saturation point. The temperature dependence of biogenic VOC emissions can influence all categories of biogenic VOC emissions because temperature affects all biochemical reactions, including those involved in the photosynthesis process [6]. The emissions of monoterpene and some oxidized VOCs are sensitive to changing moisture conditions, often increasing during and after rain events [7]. Biogenic VOC emissions are also influenced by seasonal factors such as the outbreak, growth, aging, and loss of foliage. Emissions are known to begin 2–4 weeks after budburst [8].

In the Earth's atmosphere, volatile organic compounds (VOCs) originating from both natural sources, such as plants and other living organisms, and human activities, undergo various physical and chemical transformations that can alter their characteristics [9].

Various sampling techniques and methods have been proposed for the analysis of VOCs, depending on the complexity and variability of organic vapors in the air, mainly developed for pollution surveillance [10]. Commonly employed methods for sampling biogenic volatile organic compounds (BVOCs) include the use of canisters or air sampling bags (such as Tedlar, Teflon, and Melinex) and enclosure techniques [11]. These techniques allow for the capture of a specific volume of air. However, they come with significant drawbacks, including their substantial cost and the inability to effectively preserve BVOCs [12] without altering the sample [13]. An alternative way for sampling is the use of passive sorbent or sorbent tubes [14], especially if coupled with a forced controlled flux. The substances need to undergo desorption before being analyzed using gas chromatography, employing various detectors such as mass spectrometry (MS) or flame ionization (FID) [15]. This type of sampling enables precise spatial and temporal measurements using cost-effective technology without imposing external stress on plants.

This study outlines the advancement of a novel analytical method, from sampling to analysis, for tracking volatile substances on the San Domino Island, a part of the Tremiti archipelago. The monitoring of BVOC had not previously been conducted on the island or in its vicinity, making this research the first investigation of such a unique environment. San Domino Island, spanning only 2.1 km^2^, boasts a rich diversity of vegetation that thrives in close proximity to the sea, thus experiencing its direct influence. The surveillance includes an accurate description of the vegetation to investigate their influence on the amounts of VOC, including the analysis of marine spray using cellulose filters for atmospheric particulate analysis.

Sorbent and multibed sorbent tubes are selected for BVOCs studies due to their high sampling versatility. They are compatible with both non-polar and polar compounds present in the air and can be easily stored. The tubes are made of Granular Activated Carbon (GAC) that is simple and cost-effective material. GC-MS or GC-FID, commonly available instruments, were used for the VOC analysis; Ion Chromatography (IC) was used to analyze the marine spray.

## 2 Experimental materials and methods

### 2.1 Sampling

A series of botanical surveys, described in Table S1.1 of the supplementary materials, was carried out throughout the entirety of San Domino Island. These surveys aimed to pinpoint the most characteristic and appropriate sampling locations for the analysis of soil and air components. The chosen sites are identified as “Relief ID.1 to Relief ID.4”. Once the sites were identified, samples were collected using two different types of adsorbent materials (*Uniphos coconut shell Granular Activated Carbon* and PerkinElmer sampling tubes filled with Carbotrap 300® by Supelco). In any of these places, both soil and air VOCs were sampled. The sampling procedures differ for soil and air as described in the following sections. The collected samples were analyzed using advanced laboratory techniques to provide valuable insights into the environmental characteristics and quality of the San Domino Island. Calibrated pumps, equipped with constant flow rates controller, were used for sampling with the two types of tubes loaded with different adsorbent materials. This sampling technique can be described as “medium-continuous”, as it involves the continuous storage of an air volume proportional to the preset flow rate over a predetermined duration.

Air and soil samples were collected in July 2020 and May 2021, respectively, whereas the Sea Spray Aerosol was investigated in September 2020.

#### 2.1.1 Air sampling

Air sampling was accomplished using a portable pump and sorption tubes filled with two types of adsorbent materials: GAC and Carbotrap 300. This method ensures a consistent and uninterrupted airflow, which is then used to calculate the concentration related to the total volume. Due to the very low concentration of VOCs in the ambient air, it needs to optimize both the flow rate and the withdrawal duration. After conducting several trials, the optimal sampling conditions were established at a flow rate of 0.2 L/min for a duration of 3 h.

#### 2.1.2 Soil emission sampling

The two types of adsorbent materials were used uniformly to indirectly measure soil emissions through a dynamic flow chamber. The flow chamber is a useful instrument for estimating the emission of gases/vapors (mass per unit of surface in a unit of time) originating from the ground at the designated sites. It consists of an overturned container, when sealed against the surface from which the flow is being estimated, accumulates a specific volume of air. Within this volume, the concentration of BVOCs is measured.

The dynamic flow chamber is a circular box open at its lower base, where an input flow (Qin) of 5 L/min controlled flow of carrier gas (dry and purified air) is introduced (Fig. S2.1). The input flow acts as a mixer and carrier for the flux of gases/vapors coming from the subsoil, and the output flux exits through a vent (Qs) and a sampling port (Qcamp) at a rate of 0.5 L/min. The exiting flux Qcamp from the chamber is subsequently sampled and aspirated using a suction pump and then analyzed in the laboratory.

The sampling of the output flux commences once the stationary condition beneath the chamber is achieved and continues for a duration of 3 h.

#### 2.1.3 Botanical surveys and sampling data

The initiation of the sampling campaign and the selection of specific sites were facilitated by the identification of plant species at various locations on San Domino Island in July 2020. The surveys are based on the study of the vegetation, this was carried out through the phytosociological method of the Sigmatist School of Braun Blanquet (1928). This method involves classical phytosociological study, complemented with contemporary dynamic and ecological interpretations [16,17].

This study revealed a sort of zoning pattern of the vegetation and the undergrowth of the pine forest, based on factors such as exposure and distance from the sea. The zone closest to the marine influence has a high presence of juniper, the narrow innermost strip with a higher degree of humidity has significant quantities of Leccio. The outermost strip varies according to its exposure; on the NW side, exposed to the cold and constant winds, we have a large presence of juniper in a garrigue shrub structure. Conversely, on the SE side dominates the typical formation of *Pinus halepensis* and the constant presence of *Pistacia lentiscus* with *Rosmarinus officinalis* in the openings of the forest folia. The results of the six botanical surveys conducted are compiled in Table S1.1.

**Fig. 1:**
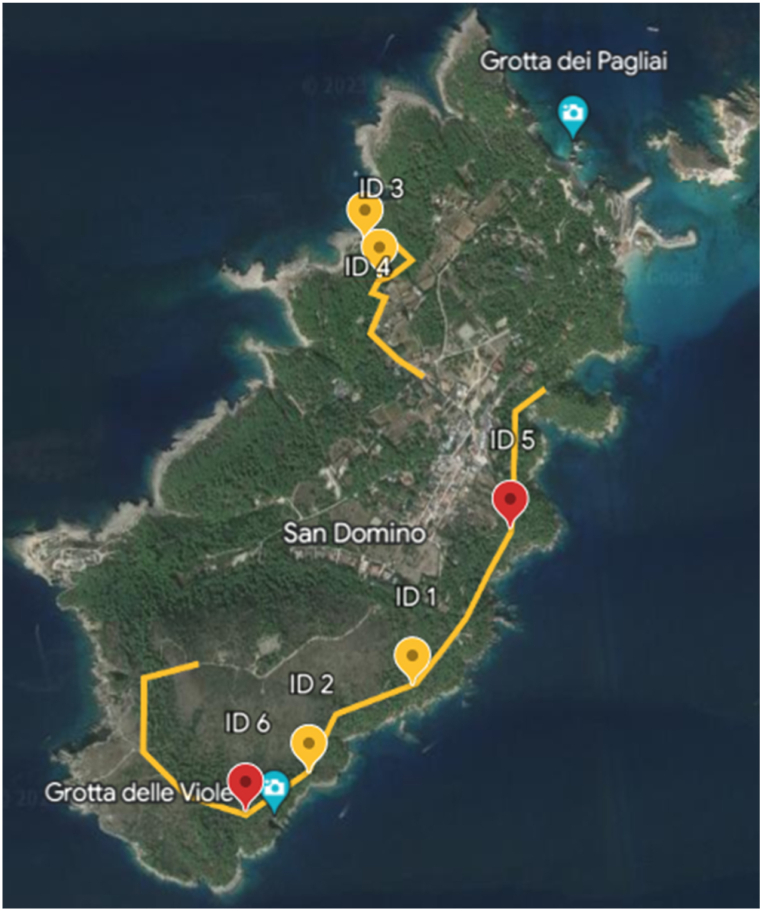
San Domino Island view with all botanical surveys from ID.1 to ID.6 (red and yellow points), sampling sites form ID.1 to ID.4 (Yellow points). The yellow line represents the trekking routes of the island.

Specifically, four out of the six conducted surveys, which exhibited the most significant differences, were selected. They differ from each other in terms of position, species, exposure to wind, and distance from the sea. The survey locations consist of two on the south side along the perimeter pathway (south) and two on the north side, as indicated in Fig. 1 with their corresponding coordinates provided in Table 1. Both highlighted paths represent two green walks accessible to everyone around the island, providing an optimal environment for engaging in forest therapy activities.

**Table 1:** Description of each sampling site. ID. and Exposure respectively means the relief IDentification and the Exposure side (S means South, N means North, W means West, E means East). The most abundant species are defined from botanical relief.

ID.	Coordinates		Exposure	Location	Most abundant species
	Long °E (gg pp ss.dd)	Lat °N (gg pp ss.dd)			
ID.1	15°29′20.3″	42°06′32.0″	S/SE	Grotta del Sale	*Rosmarinus officinalis*
ID.2	15°29′06.8″	42°06′24.1″	SE	Cala delle Roselle	*Pistacia lentiscus*
ID.3	15°29′16.3″	42°07′08.6″	W/NW	Cala Tramontana A (internal area)	*Pinus halepensis*
ID.4	15°29′14.6″	42°07′12.0″	NW	Cala Tramontana B (external area)	*Juniperus turbinata, Helichrysum italicum,* *Myrtus communis*

GAC sampling tubes were used for qualitative analysis at the four selected sites under consideration. The identical tubes were used in a semi-quantitative monitoring of α-pinene and D-limonene at sites ID.2 and ID.3 to ascertain their daily emission. In three of the four sites, parallel samples were collected using Carbotrap 300 tubes to compare the qualitative results with those obtained with the GAC tubes. Soil emission was measured in all sites except the relief ID.4 due to its arid nature, primarily covered with gravel. The air sampling carried out in May 2021, with temperatures ranging from 18 °C to 23 °C, is described in Table 2.

**Table 2:** Sampling of BVOC with Activated Carbon (GAC) and Carbotrap 300 (TD) in San Domino Island. The samples in bold were sampled with GAC, while the samples in italic was taken with Carbotrap300.

Relief ID species Time range/Type of sampling			Relief ID. 1 *Rosmarinus officinalis*	Relief ID. 2 *Pistacia lentiscus*	Relief ID. 3 *Pinus halepensis*	Relief ID. 4 *Helichhrysum italicum* *Juniperus turbinata* *Myrtus communis*
h.6:00–9:00	Air	GAC	Sample n. **70**, 71, 72, 73	Sample n. 80	Sample n. 88, 89	–
		TD	–	–	Sample n. *53*	–
	Soil	GAC	Sample n. **86**	Sample n. 81	Sample n. **75**	–
		TD	–	–	Sample n. 52	–
h. 11:00–14:00	Air	GAC	–	Sample n. **82**	Sample n. 76	–
		TD	–	Sample n. *55*	–	–
	Soil	GAC	–	Sample n. **83**	Sample n. 77	–
		TD	–	Sample n*. 54*	–	–
h. 17:00–20:00	Air	GAC	–	Sample n. 84	Sample n. **78**	Sample n. **87**
		TD	–	–	–	Sample n. *56*
	Soil	GAC	–	Sample n. 85	Sample n. 79	–
		TD	–	–	–	–

**Table 3:** Qualitative result of Activated Carbon (GAC) and Carbotrap300 (TD) analysis. KIc Kovats Index calculates for HP5 column, KIa Kovats Index found in Adams (2017), KIn Kovats index found in NIST (2017), KIo Kovats index from another library.

						ID.1 - AIR	ID.1 - SOIL	ID.2 - AIR		ID.2 - SOIL		ID.3 - AIR		ID.3 - SOIL		ID.4 - AIR	
						GAC	GAC	GAC	TD	GAC	TD	GAC	TD	GAC	TD	GAC	TD
	SE-GC			TD-GC		70	86	82	55	83	54	78	53	75	52	87	56
Compound name	KIc	KIa	KIn	KIc	KIo	%	%	%	%	%	%	%	%	%	%	%	%
1,3,8-*p*-Menthatriene	–	–	–	1166	–	–	–	–	–	–	–	–	0.21	–	–	–	–
1-Hexanol,2-ethyl	–	–	–	1076	–	–	–	–	–	–	–	–	–	–	21,05	–	–
1-Octanol	–	–	–	1117	–	–	–	–	–	–	0.90	–	–	–	1,46	–	–
2-Heptenal	–	–	–	1014	–	–	–	–	–	–	0.55	–	–	–	0,56	–	0,49
3-Carene	1004	1011	1011	1033	1022	1.57	53.66	–	0.79	4.86	1.89	–	3.04	1.97	3,86	7,82	40,92
4-pentenal	–	–	–	916	–	–	–	–	–	–	0.20	–	–	–	0,17	–	–
allo-Ocimene	–	–	–	1151	–	–	–	–	–	–	–	–	0.09	–	–	–	–
α-Pinene	939	939	937	954	950	15.72	–	2.68	57.70	53.09	8.79	64.46	46.40	58.88	12,75	10,21	0,01
α−Terpinene	1027	1017	1017	1043	1030	–	–	–	0.37	–	0.37	–	0.30	–	–	1,43	0,63
α−Thujene	932	930	927	944	944	3.30	–	–	12.69	–	1.87	–	0.21	–	0,37	1,54	7,63
β−cis-Ocimene	–	–	–	1049	–	–	–	–	–	–	–	–	0.11	–	–	–	0,10
β−Myrcene	992	990	989	1002	1005	–	–	–	–	–	7.17	10.71	36.99	2.13	7,64	–	–
β−Phellandrene	–	–	–	1061	1051	–	–	–	0.38	–	0.47	–	0.44	–	0,36	–	22,69
β−Pinene	981	979	978	1000	–	11.97	–	–	5.29	6.83	–	3.92	Trace	5.97	–	6,23	–
Camphene	954	954	950	976	972	12.37	–	–	–	3.26	0.25	–	1.54	0.79	0,22	1,15	0,16
Camphor	1136	1146	1144	–	–	–	–	–	–	–	–	–	–	–	–	2,25	–
Decanal	1209	1201	1204	1255	–	4.41	15.68	16.83	–	–	2.38	1.79	Trace	1.37	0,22	4,36	Trace
Decanol	–	–	–	1522	–	–	–	–	–	–	0.97	–	–	–	22,69	–	1,36
D-Limonene	1033	1029	1024	1053	1054	5.83	13.24	42.83	3.79	17.13	19.71	10.99	3.40	16.26	1,34	31,38	4,24
Dodecanal	–	–	–	1458	–	–	–	–	–	–	2.90	–	–	–	1,22	–	–
endo-Borneol	1158	1169	1169	–	–	–	–	–	–	–	–	–	–	–	–	Trace	–
Ethylbenzene	–	–	–	888	–	–	–	–	–	–	Trace	–	–	–	Trace	–	0,02
Eucalyptol	1036	1031	1031	1065	–	28.30	–	–	–	–	–	–	0.24	Trace	–	2,11	Trace
Fenchene	–	–	–	969	–	–	–	–	–	–	–	–	Trace	–	–	–	0,15
Fencocamphorone	–	–	–	1181	–	–	–	–	–	–	0.34	–	–	–	–	–	–
γ−Terpinene	–	–	–	1076	–	–	–	–	1.16	–	22.27	–	0.22	–	–	–	–
Heptane, 3-methylene	–	–	–	794	–	–	–	–	Trace	–	9.99	–	0.08	–	9,71	–	0,01
Hexanal	–	–	–	841	840	–	–	–	–	–	0.59	–	–	–	0,51	–	–
Hexanal, 2-ethyl	–	–	–	994	–	–	–	–	–	–	Trace	–	–	–	2,24	–	–
Linalool	–	–	–	1143	–	–	–	–	–	–	–	–	0.40	–	–	–	0,11
*m*-Cymenene	–	–	–	1117	–	–	–	–	–	–	–	–	0.13	–	–	–	–
Nonanal	1107	1100	1104	1151	1151	4.82	–	17.21	–	–	3.80	1.41	–	–	5,14	1,18	–
Octanal	–	–	–	1048	1039	–	–	–	–	–	1.13	–	–	–	1,35	–	–
*o*-Cymene	–	–	–	1017	–	–	–	–	–	–	–	–	0.44	–	–	–	–
*o-*Xylene	–	–	–	927	–	–	–	–	–	–	0.61	–	Trace	–	0,57	–	–
*p*-Cymene	1029	1024	1024	1057	1059	2.74 (54)	–	10.90	–	2.45	0.74	2.01	Trace	4.70	Trace	23,50	3,27
*p*-Cymenene	–	–	–	1112	–	–	–	–	–	–	–	–	0.64	–	–	–	–
*p-*Xylene	–	–	–	896	892	–	–	–	–	–	0.48	–	–	–	0,45	–	–
Sabinene	–	–	–	997	991	–	–	–	7.46	–	4.41	–	–	–	2,24	–	8,35
Santolina triene	–	–	–	921	–	–	–	–	–	–	–	–	Trace	–	Trace	Trace	–
Terpinolene	–	–	–	1110	–	–	–	–	–	–	–	–	0.41	–	–	–	–
Thuja-2,4 (10)-diene	–	–	–	975	–	–	–	–	–	–	–	–	0.44	–	–	–	0,13
Toluene	–	–	–	792	791	–	–	–	1.67	–	Trace	–	Trace	–	Trace	–	0,14
trans-Mentha-2,8-diene	–	–	–	993	–	–	–	–	–	–	–	–	0.17	–	–	–	–
Tricyclene	920	–	918	896	892	–	–	–	–	1.71	–	–	Trace	–	–	–	–
Undecanal	–	–	–	1358	–	–	–	–	–	–	0.81	–	–	–	0,46	–	–
		% of identification				91,02	82,58	90,44	91.30	89.33	93.58	95.29	95.90	92.06	96.59	93.16	90.41
		n. of components				10	3	5	11	7	28	7	29	9	27	15	20

#### 2.1.4 Sea spray aerosol (SSA) emission sampling

In September 2020, air sampling was conducted to analyze SSA and evaluate its impact, considering the prevailing winds on San Domino Island. During sampling, the 0.8 μm MCE membrane filter is supported by a MegaSystem sample holder. All sampling was carried out for a duration of 2 h at a flow rate of 3.5 L/min at the sites ID.1, ID.2, ID.3, and ID 4. Detailed sampling conditions are reported in Table 4.

**Table 4:** Quantitative results of anions for Sea Spray Aerosol related to the sampling conditions.

Anion Sample	Cl^−^(μg/m^3^)	I^−^(μg/m^3^)	F^−^(μg/m^3^)	NO_2_^−^(μg/m^3^)	NO_3_^−^(μg/m^3^)	SO_4_^=^ (μg/m^3^)	SAMPLING CONDITIONS		
							Exposure	Prevailing wind (kts)	Wave (m)
32 – ID.4	54.3	0.023	0.4	2.0	24.3	48.8	NW^1^	NW (7 kts)	0.5
33 – ID.3	12.6	0.006	<LOD^2^	3.3	22.5	18.1	W/NW^3^	NW (5 kts)	0.4
34 – ID.4	12.7	0.005	<LOD	1.3	24.8	17.1	NW	E^4^ (12 kts)	0.2
35 – ID.3	16.5	0.005	0.9	2.9	30.5	22.4	W/NW	NW (5 kts)	0.1
36 – ID.1	12.2	0.006	0.5	1.6	21.6	16.9	S/SE	N (14 kts)	0.9
37 – ID.2	14.6	0.007	<LOD	1.4	18.8	20.2	SE^5^	NW (15 kts)	1.3
47– ID.1	78.5	0.010	0.5	5.5	28.1	29.2	S/SE^6^	SW^7^ (20 kts)	1.4
48– ID.1	88.6	0.046	0.5	<LOD	38.7	32.0	S/SE	SW (24 kts)	1.5
49– ID.1	47.4	0.026	<LOD	1.5	45.8	31.9	S/SE	N (4 kts)	0.3
50– ID.2	42.3	0.020	<LOD	<LOD	39.2	33.4	SE	N (5 kts)	0.4

1 “NW” means North-West.

2 “<LOD” means minor than Limit Of Detection of the method.

3 “W/NW” means West/North-West.

4 “E” means East.

5 “SE” means South-East.

6 “S/SE” means South/South-East exposure on the island.

7 “SW” means South-West direction of the prevailing wind.

### 2.2 Equipment and analysis

The comparison and optimization of the experimental procedure for the determination of BVOCs in open air are described below. We conducted a performance comparison between two absorbent materials: Granular Activated Carbon (GAC) coupled with solvent extraction, and Carbotrap 300 from Supelco used with thermal desorption. The latter method has already been evaluated and validated as a method by UNICAM's Analytical Chemistry research group and is applied in the same way [18]. Activated Carbon is usually used for the monitoring of polluted sites, in this study, we evaluate its applicability for BVOC sampling. Absorption is contingent on physical and chemical phenomena, the type of adsorbent, the nature of the organic compounds, temperature, suction flow, and humidity levels. Thoughout the experimental phase, we implemented the Quality Assurance protocols, incorporating preventive measures to minimize potential systematic errors. Regular instrument calibrations were conducted to ensure alignment with reference standards. Systematic internal quality checks were carried out to verify measurement accuracy and to identify any anomalies. In terms of Quality Control, we consistently monitored data repeatability, reproducibility, and calibration, including cross-checks among operators. Detailed QA and QC is described in supplementary materials (section S3).

#### 2.2.1 Carbotrap 300 and TD-GC-MS analysis

PerkinElmer sampling tubes filled with Carbotrap 300® (Supelco) were used for the sampling of air on site by means of a suction pump that helps to concentrate the volatile analytes on the sorbent material, where they stabilized and were retained for subsequent TD-GC-MS laboratory analyses. These analyses were carried out in accordance with the ISO 16017–1:2002, CEN/TS 13649:2015, and EPA TO-17 official methods for TD analysis of VOC/SVOC, as already applied for BVOCs.

Thermal Desorption analyses were performed with TD PerkinElmer TMX350 coupled with GC Shimadzu GCQP2010 with capillary column Rtx-624SilMS (30 m × 0.25 mm x 1.4 μm, fused silica column, Restek) and MS Shimadzu GCMS QP2010 Plus for mass spectrometry analysis.

The desorption process takes place within a temperature range of 250 °C–325 °C, utilizing helium as the carrier gas at a desorption flow rate ranging from 30 ml/min to 50 ml/min. This flow transports the BVOCs into a cold trap set at −10 °C, causing their condensation. Subsequently, the BVOCs are moved directly into the GC column over a temperature range of 250 °C–350 °C. The GC temperature ramp starts at 45 °C for 5.5 min then increases at the rate of 5 °C/min until reaching 170 °C, followed by a further increase at a rate of 20 °C/min until 300 °C, and held for 8 min (total of 45,00 min). These conditions were used for all TD-GC-MS analyses. A C7–C20 alkane mix was used to calculate the Kovats Index (KI).

#### 2.2.2 Activated carbon GAC and SE-GC-FID/MS analysis

Activated carbons were subjected to two extractions using 0.5 ml of distilled n-hexane; the recovery of the extract was carried out using a glass pipette with cotton as a filtering agent. The solution is analyzed with GC-FID with HP-5 column and GC-MS from Agilent with HP5-MS column. The GC temperature ramp and the Kovats Index (KI) calculus are detailed in section 2.2.1.

GC-MS is employed solely for compound identification, whereas all qualitative and quantitative considerations rely on GC-FID analyses. Semi-quantitative analysis was conducted specifically for activated carbon, focusing on limonene and a-pinene, the most abundant compounds in the samples. This analysis followed the calibration of the analytical method, as described in supplementary materials (section S3). The response factor (RF) of the selected molecules can be applied to other molecules within the same class [19]. This assumes that molecules with similar structure possess comparable response factors, allowing for a semi-quantitative evaluation.

#### 2.2.3 Cellulose filter and IC analysis

Marine spray was collected using equipment designed for gravimetric dust measurement. Sampling and analysis were carried out following UNI EN ISO 10304–1:2009, the NIOSH 7906/07/08:2014, and APAT 4020 validated methods (Determination of fluoride anions, chloride, nitrite, bromide, nitrate, phosphate, sulfate by ion chromatography).

The MCE membrane filters were extracted with 10 ml of an aqueous solution of sodium carbonate/sodium hydrogen carbonate; the same solvent was used as the eluent in the ion chromatography measurements. The chromatograph used is Metrohm Eco IC with column Metrosep A Supp 5–250/4.0. The analysis involves a chromatographic run of 48 min with a flow rate of 0.700 ml/min. The quantification of I^−^ was carried out with Thermo Scientific ICP-OES iCAP PRO.

## 3 Results and discussion

### 3.1 Evaluation of solvent extraction (SE)

The standard procedure UNI CEN/TS 13649 used for analyzing IPA and BTEX, necessitates the transfer of the material containing VOCs to a vial, followed by the addition of a solvent with high VOC stability, such as carbon disulfide. After stirring to enhance the desorption of the VOCs, the resulting clear solution is injected into the GC system. Two different solvents were evaluated, based on the standard analysis conditions for pollutants adsorbed on activated carbon. Carbon disulfide, a commonly used solvent, was compared to the safer alternative solvent, n-hexane, due to concerns its flammability, irritability, toxicity to the central nervous system, and harmful effects on reproduction. To evaluate the optimal extraction conditions, a reference sample was prepared by conducting a 12-h sampling on a *Rosmarinus officinalis* plant under controlled conditions. This reference sample was used as a benchmark to assess the efficiency of different extraction methods. The methods were compared to maximize the extraction yield and the purity of the extracted compounds. We partitioned activated carbons, used for sampling, into two vials (200 mg each), one of them extracted with 0.5 ml of CS_2_ and the other with 0.5 ml of n-hexane (minimum amount necessary to cover activated carbons and limit the dilution). Hexane showed the highest extraction quality, indicating that it is the most suitable solvent for this sample. The best performance of hexane is shown in Fig. S4.1.

An optimization study was conducted to investigate a two-step extraction procedure using hexane. The extraction was carried out twice in the ultrasonic bath for 30′ mint with 0.5 ml of n-hexane. The two extracts were combined in a high recovery vial and then reduced to a volume of 40 μl with a nitrogen flux. To ensure method reproducibility, the vial was weighed once the volume reached 40 μl; by using the density of n-hexane (d = 0.66 g/cm^3^), the extract amount was determined to be 0.0264 g. Fig S4.2 shows higher extraction quality obtained with this two-step procedure.

Samples must be analyzed within one week and stored at 4 °C to avoid degradation of the sample analytes as described in paragraph 3.2.

### 3.2 Terpenes degradation

Terpenes are highly susceptible to degradation through oxidation reactions, which has been observed in studies where adsorbent materials showed degradation within the first week after sampling, persisting for up to one month [20]. The GC-FID analysis of a 12-h Rosmarinus officinalis reference sample permits the observation of degradation products after three months of storage at room temperature, as shown in Fig. S5.1. These compounds primarily originate from high molecular weight terpenes and are result from chain breakage, leading to the formation of alkanes, branched alkanes ranging from C10 to C19, aromatic rings as xylenes, and alkylated benzene rings as C_6_H_6_(CH_3_)_3_.

In addition, the degradation of a limonene standard adsorbed on activated carbons was evaluated after 4 days, 1, 2, and 3 months of storage at room temperature, employing the aforementioned double extraction procedure.

The results show a decrease in limonene percentage: 93 % in the first extraction, 86 % in the second extraction, 80 % in the third extraction, and 72 % in the fourth extraction. correspondingly there was an increase in the formation of degradation products, primarily observed in the third extraction, as shown in Fig. S5.2) Degrading reactions accelerate, particularly with higher room temperatures and prolonged storage periods. Consequently, it is necessary to store the samples at 4 °C immediately after sampling and analyze them within a week to preserve sample integrity.

### 3.3 Qualitative analysis

Qualitative identification of compounds found in analyzed samples by GC-FID and GC-MS, conducted based on the Kovats retention index, using values found in the literature [21], and the comparison of the fragmentation pattern of molecules using the NIST14 mass spectrum library. KI was calculated by analyzing the alkane mixture under the same analysis conditions. The qualitative findings remain consistent across sampling carried out at different times of the day at the same site (samples 70,71,72,73 of relief ID.1, Table 1). The results by sampling type are shown in Table 3 and S6.1.

It is feasible to discern the relative abundance of specific components at each sampling site. Qualitative analyses show that the sites with a predominant presence of *Rosmarinus officinalis (ID.1)* and *Helichhrysum italicum, Juniperus turbinata* and *Myrtus communis* (*ID.4*) emit a more diverse range of BVOCs.

The measurements with GAC in the site ID.4 show the abundance of α-thujene (1.54 %), α-pinene (10.01 %), β-pinene (6.23 %), camphene (1.15 %), 3-carene (7.82 %), *p*-cymene (23.50 %), D-limonene (31.38 %) and camphor (2.25 %). These are the compounds mostly emitted by *Myrtus communis* and found in its essential oil [22].

Measurements with GAC in site ID.1 indicate the abundance of α-pinene (15.72 %), β-pinene (11.97 %), camphene (12.37 %), D-limonene (5.83 %), eucalyptol (28.30 %), *p*-cymene (2.74 %); these compounds are usually found in *Rosmarinus officinalis* plants [23].

*Pinus halepensis* and *Pistacia lentiscus*, the most abundant species all over the Island, are characterized by the 3-carene, α-pinene, β-myrcene, β-pinene, D-limonene and *p*-cymene, as identified in the samples from sites ID. 2 and ID. 3. Similar patterns were observed in both soil and air from these locations.

TD-GC-MS analysis enhances differentiation in terpene emissions, providing distinctive characteristics to different sites.

**Fig. 2:**
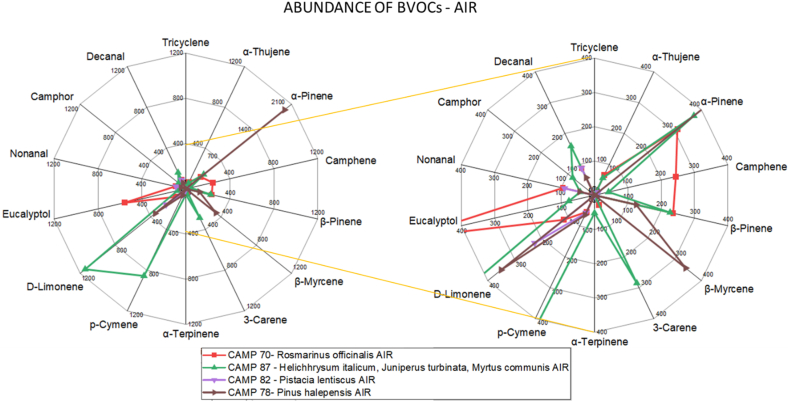
Abundance of different BVOCs in air analysis for representative sampling in each botanical relief. An enlargement of the central area of the graph is shown on the right-hand side.

**Fig. 3:**
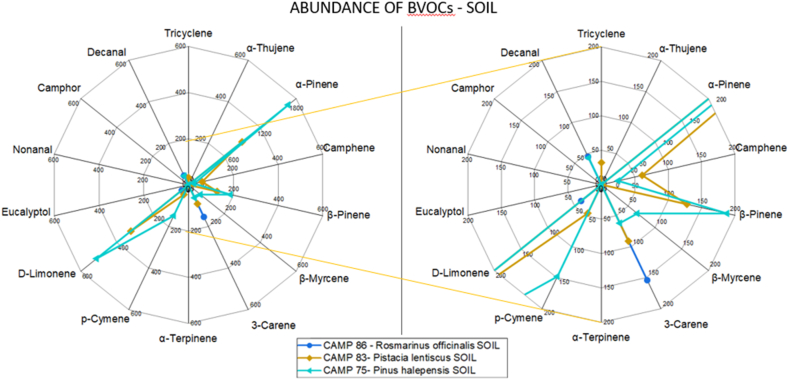
Abundance of different BVOCs in soil analysis for representative sampling in each botanical relief. On the right is an enlargement of the central area of the graph.

Fig. 2, Fig. 3 show the comparison between air and soil analyses, respectively, with targeted sampling for *Pinus halepensis* (ID.3), *Pistacia lentiscus* (ID.2), *Rosmarinus officinalis* (ID.1), and *Helichhrysum italicum, Juniperus turbinata, Myrtus communis* (ID.4).

Fig. 2 shows that the composition of sites ID.2 and ID.3 is very similar as they are graphically overlapping, confirming the coexistence of the two species in their respective sampling sites. However, a difference can be observed for ID.1 and ID.4 from the moment when they exhibit different abundances of eucalyptol and camphene.

Fig. 3 shows the soil emission of one sample for every relief except ID.4 because of its rocky nature. A substantial compositional identity can be observed for the four sites, probably due to a predominance of degraded *Pinus halepensis* needles in the soil. Furthermore, a correlation between air and soil analyses of *Pinus halepensis* can also be noted, confirming what is highlighted by the figures.

**Fig. 4:**
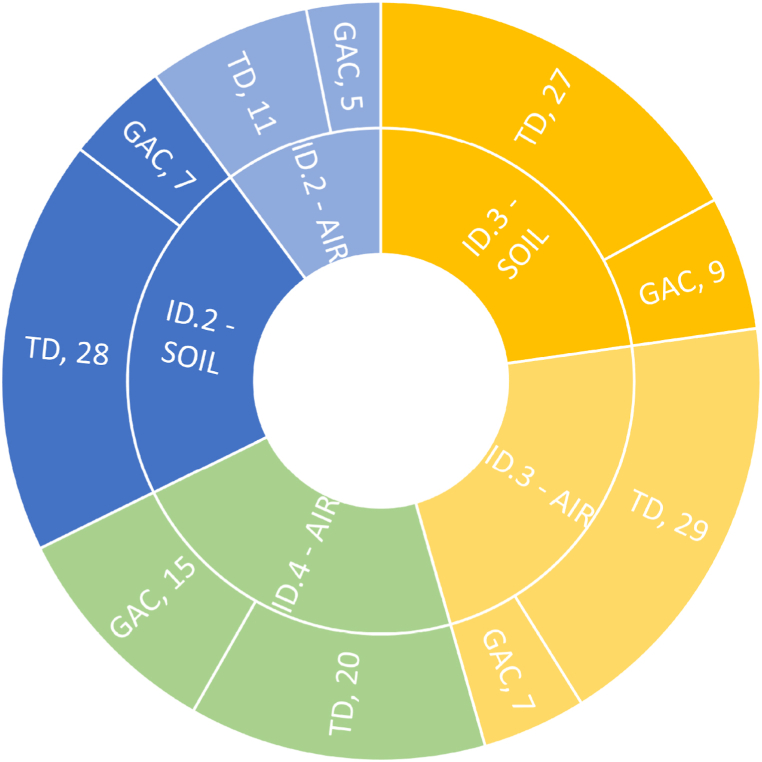
Comparison of TD and GAC. In the outer ring the number of compounds follow the acronym of the technique. Different colors are associated to the relief while pale color indicates air samples and vivid color the soil samples.

In Fig. 4, a comparison between the two analytical techniques is presented in terms of the number of components detected in each type of sampling (GAC and TD).

Note that thermal desorption (TD) combines with Carbotrap300 tubes. The synergy of an enhanced absorber with a highly sensitive extraction technique (TD-GC-MS) allows the detection of even trace components, offering a comprehensive qualitative insight. However, GAC sampling tubes yield valuable results for site characterization in a cost-effective manner, offering a more economical alternative to techniques involving thermal desorption.

A Principal Component Analysis (PCA) was conducted to characterize the investigated system. The results obtained revealed that the first three principal components account for over 70 % of the variability within the system, increasing to 99 % with the inclusion of the first nine components (Fig. S7.1). These findings highlight the presence of substantial variability within the system. The Score Plot (Fig. S7.2), despite a variability of 63.9 %, demonstrates a correlation between different TD analyses, which is also evident in the GAC analysis. Notably, a correspondence is observed between the analyses of air and soil at the site abundant with Pistacia lentiscus (Relief ID.2) and Pinus halepensis (Relief ID.3), indicating a blend of Biogenic Volatile Organic Compounds (BVOCs) owing to the coexistence of these species (Fig. S7.3). Nevertheless, it is crucial to acknowledge that the use of two different techniques did not always result in perfect correspondence between the same analyses, indicating variations in efficiencies and adsorption capacities of the employed adsorbent materials (Fig. S7.4).

### 3.4 Quantitative analysis

#### 3.4.1 Semi-quantitative analysis of BVOCs and daily trend

Quantification was carried out as described in section 2.2.2 for air and soil samples, repeated three times within the same day, corresponding to the sites ID.3 and ID.2 (*Pinus halepensis* and *Pistacia lentiscus*).

**Fig. 5:**
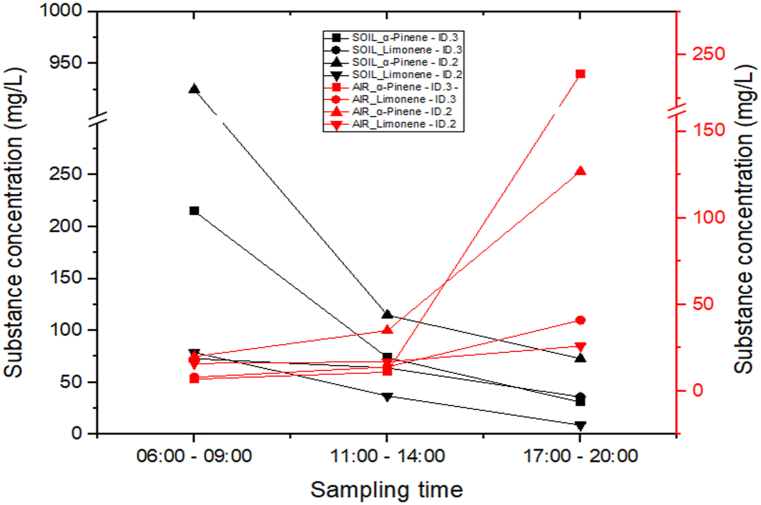
Concentration (mg/L) of α-pinene and D-limonene for soil (black) and air (red) measured at site ID.2 and ID.3.

The semi-quantitative analysis of the activated carbon made it possible to verify the trend of the daily emissions both in the air and in the ground. Fig. 5 shows the results obtained with the same sampling and extraction conditions of the qualitative analysis.

The amount of α-pinene and limonene emitted from the soil exceeds the amounts detectable in the atmosphere. This disparity can be attributed to the varying distances of the emission sources from the sampling.

It is interesting to note the temporal patterns of BVOC emissions originating from different sources. The inverse correlation between α-pinene and limonene emissions suggests that different factors might influence their release from a common source. The diurnal trend of BVOC emissions from vegetation aligns with prior research findings, indicating that higher temperatures and light intensity during the day time enhance photosynthetic activity, consequently leading to higher BVOC emissions. The increased emission of BVOCs in the latter part of the day may be attributed to the accumulation of VOCs throughout the day, coupled with the influence of temperature and light on the production and release of BVOCs. It is estimated that the augmented BVOC release during daylight hours, coinciding with the zenith of light intensity and temperature, contributed to higher daytime concentrations [24].

A comparable trend has been observed in the northern Italian Apennines [25] where the peak of emissions occurs between 2 p.m. and 4 p.m., followed by a decline in the late afternoon.

On the other hand, the emission from soil shows a different temporal pattern, an abundant emission in the morning with a decreasing trend. In the morning, as solar radiation intensifies, the soil undergoes warming, leading to significant evaporation of the moisture accumulated overnight. This phenomenon creates a shallow, unstable boundary layer. Currently, BVOC emissions from trees are still relatively low due to the low levels of radiation and temperature. However, BVOCs can diffuse within a confined vertical layer, resulting in their accumulation near the ground at relatively high concentrations.

In all analyzed samples the detected substances showed the consistent general trend. This uniform behavior of the target compounds across diverse samples is a valuable finding, indicating a probable common origin or influence by similar environmental factors for the analyzed substances. Moreover, the consistent trend observed in the target compounds highlights the robustness and reproducibility of the analytical method used in this study. The spot sampling allowed a limited number of samples, to address this limitation, the Ion Science TIGERLT portable total VOCs detector was used. This portable gas detection instrument allows rapid and accurate measurements of a wide range of volatile organic compounds (VOCs). The use of the TIGERLT during the samplings permits the evaluation of the total VOC emission trend (Fig. S8.1) and is useful for a general screening and immediate monitoring.

#### 3.4.2 Quantitative analysis of anions of SSA

Atmospheric particulate matter (PM) comprises several chemical compounds, with a predominant presence of inorganic ions such as fluoride, chloride, calcium, magnesium, sodium, potassium, ammonium, nitrate, and sulfate (F^−^, Cl^−^, Ca^++^, Mg^++^, Na^+^, K^+^, NH _4_^+^, NO_3_^−^, SO_4_^−^).

The presence of atmospheric particulate matter is affected by multiple factors, including the geographical location of the area, seasonal variations, and the presence of natural or anthropogenic pollution sources. In the Central Mediterranean region, a semi-enclosed area, elevated levels of particulate matter are observed, stemming from sources both within the vicinity and from distant locations. Quantitative results for the considered anions are shown in Table 4, sampling, and analysis procedure are described in paragraph 2.1.4.

Fresh sea-salt aerosols are primarily composed of unaltered bulk seawater, characterized as a sodium chloride and magnesium sulfate brine with a pH of 8.1 [26]. This composition applies mostly to the larger size of sea-salt aerosol particles, which dominate the overall sea-salt mass distribution. However, the composition analysis of fine marine aerosol particles is intricate due to their amalgamation with atmospheric non-sea-salt and organic particles originating from diverse sources.

NO_3_^−^ exhibits its highest concentrations in the coarse mode fractions due to nitric acid displacing Cl^−^ within sea salt aerosols [27,28]. The NO_2_^−^ ion can originate from natural sources, such as lightning, as well as from anthropogenic sources. However, the analysis performed in this study lacks the capability to distinguish between the two types of sources. It is well-known that anthropogenic sources such as vehicle emissions, industrial activities, and power generation are major contributors to NO_2_^−^ concentrations in urban environments. Nevertheless, it is crucial to note that natural sources, such as soil emissions and biogenic processes, can also play a significant role in NO_2_^−^ concentrations, particularly in rural areas.

Sulfate (SO_4_^2−^) ranks as the second most prevalent anion after chloride in the contemporary ocean and serves as an energy source for sulfate-depleting prokaryotes (SRPs), frequently found in organic-rich sediments, and plays an important role in the decomposition of organic matter [29]. Sulfates are significant compounds in the formation of aerosols in the marine atmosphere and are probably the most abundant particles in terms of aerosol number. They contribute to the aerosol optical depth of the marine atmosphere and are a significant source of cloud condensation nuclei [26].

Atmospheric iodine is mainly derived from the oceans, which contain approximately 70 % of the Earth's surface inventory of natural iodine [30]. Several iodine-containing species have been demonstrated to be released from the marine environment, among which methyl iodide (CH_3_I), diiodomethane (CH_2_I_2_), chloroiodomethane (CH_2_ICl), bromoiodomethane (CH_2_IBr) and I_2_ are considered the primary sources of marine atmospheric iodine [31].

Gas-to-particle conversion processes are the dominant source of iodine in marine aerosols. Iodide ions in seawater are oxidized to elemental iodine, which volatilizes into the atmosphere and is subsequently returned to the soil through rainfall, completing the cycle. Iodine is a key trace element in continental food chains, and its major global source is oceanic surface gas emissions of iodine-bearing molecules to the atmosphere. Following atmospheric chemical processing, the iodine-bearing products are incorporated into marine aerosol particles that become airborne. These aerosol particles act as carriers of iodine, transporting it from the ocean to the continents [32]. Iodine has a unique geochemistry among the halogens due to its considerably higher ease of volatilization in both inorganic and organic forms compared to chlorine and bromine. This property of iodine makes it stand out among the halogens.

The primary source of chlorine-containing gases in the troposphere is the volatilization of inorganic Cl species from primary marine aerosols. However, sources associated with human activities also play a significant role over land [33].

Sources of gaseous and particulate fluoride (F^−^) encompass both anthropogenic and natural emissions. Anthropogenic sources of fluoride include activities such as coal burning, ceramic, brick, and cement factories [34]. Conversely, natural sources of fluoride include volcanic activity [35] and seawater spray [36].

A study on the composition of rainfall in the regions of southern Italy confirms the trend of concentration found in the air, where the major ionic species followed the sequence Cl^−^> SO_4_^2−^> NO_3_^−^> F^−^ [37]. Sea spray aerosols (SSAs) have profound effects on climate and ecosystems. Furthermore, the presence of microbiota and biogenic molecules, produced by among others marine phytoplankton within SSAs could lead to potential human health effects. While the exposure and effects of SSAs on human health remain insufficiently studied, they are known to have beneficial effects on human health.

## 4 Conclusion

The monitoring of the BVOCs emitted in San Domino Island has enabled the identification of 13 predominant components in the air. These include tricyclene, α-thujene, α-pinene, camphene, β-pinene, β-myrcene, 3-carene, α-terpinene, *p*-cymene, D-limonene, eucalyptol, camphor and endo-borneol. They belong to the major BVOCs emitted by Mediterranean plant species [38]. These compounds are emitted by the different species on San Domino Island and from the ground, primarily covered by pine needles. The semi-quantitative analysis shows that compounds can be detected during the daytime, given the contrasting emission trends of soil and vegetation. This disparity is attributed to the influence of light and temperature on the emission rate [39]. Moreover, the winds prevailing on the island, while posing challenges to sampling efforts, ensure the continuous transport of BVOCs to various locations. From qualitative analysis, focusing on the different classes of components, it becomes feasible to compare different analytical techniques in terms of their adsorption capacity and subsequent release of analytes. The findings show that activated carbon exhibits the highest adsorption capacity for the BVOCs emitted, thereby characterizing the components in the island's forest environment. The sensitivity of activated carbons is lower in comparison to thermal desorption techniques. However, the use of activated carbons is justified by the material's cost-effectiveness, simplicity, and availability of extraction and analysis techniques. The study conducted using activated carbon vials has certain limitations, particularly in terms of sensitivity. Consequently, it may not be capable of detecting trace levels of BVOCs, unlike methods employing devices such as carbotrap300 vials with thermal desorption or SPME (Solid Phase Microextraction) fibers, unless extended sampling durations are employed.

All the detected substances are emitted by the vegetation on the island, as proved by the SPME analysis of each single species.

The results obtained in this study can be useful for touristic exploitation, offering crucial insights into the availability, spatial, and temporal distribution of VOCs on San Domino Island. This information can be utilized to promote forest therapy practices [40,41]. By analyzing VOC concentrations in different environments allows for insights into the specific compounds and concentrations conducive to human health. This analysis aids in developing strategies to utilize these compounds effectively, promoting health and well-being. These results can be used to promote further research on the BVOC and human health relationship. Moreover, they can serve as foundation for developing potential therapy approaches that harness the therapeutic properties of BVOCs to improve human health. In our case, it can have a synergistic effect with SSA as marine iodine has been studied extensively due to its essential role in human health. Its deficiency can lead to a variety of health problems, known as iodine deficiency disorders (IDD). The sea plays a significant role in the global cycle of iodine. Although iodine is primarily concentrated in coastal soils because of its dominant oceanic source, the main marine influence extends as far as 80 km in land.

## Data availability statement

Data included in article/supplementary material/referenced in article. Other data will be available on request.

## Funding

This review has no funding affiliation to declare.

## Ethics

Not applicable.

No additional information is available for this paper.

## CRediT authorship contribution statement

**Martina Fattobene:** Writing – review & editing, Writing – original draft, Visualization, Investigation, Formal analysis, Data curation. **Fabrizio Papa:** Investigation, Formal analysis, Data curation. **Raffaele Emanuele Russo:** Investigation. **Silvia Zamponi:** Writing – review & editing. **Paolo Conti:** Writing – review & editing, Visualization. **Fabio Taffetani:** Investigation, Data curation. **Adelmo Sorci:** Resources, Investigation. **Fuyong Liu:** Investigation, Data curation. **Mario Berrettoni:** Writing – review & editing, Supervision, Methodology, Conceptualization.

## Declaration of competing interest

The authors declare that they have no known competing financial interests or personal relationships that could have appeared to influence the work reported in this paper.
