# Supplementary material for: ON-SITE monitoring OF BVOCS emission in Tremiti island, Italy
Source: Heliyon. 2023 Dec 19;10(1):e23822. doi: 10.1016/j.heliyon.2023.e23822 (PMC10772626; doi:10.1016/j.heliyon.2023.e23822)
Supplement: Multimedia component 1 [file mmc1.docx]

**ON-SITE MONITORING OF BVOCS EMISSION IN TREMITI ISLAND, ITALY**

Martina Fattobene**^a^**, Fabrizio Papa**^a^,** Silvia Zamponi**^a^**, Paolo Conti**^a^**, Raffaele Emanuele Russo**^a^**, Fabio Taffetani^b^, Adelmo Sorci^c^, Mario Berrettoni**^a^**

**^a^**School of Science and Technology, Chemistry Division, University of Camerino, Via Madonna delle Carceri – ChIP, Camerino (MC) 62032, Italy.

**^b^**Dipartimento di Scienze Agrarie, Alimentari e Ambientali, Università Politecnica delle Marche, Via Brecce Bianche, Ancona (AN) 60131, Italy.

^c^Laboratorio del Ma.Re, Via A. Vespucci, Isole Tremiti (FG) 71040, Italy

^d^Zhengzhou University of Light Industry, Zhengzhou 45000, China

*Corresponding author.

E-mail address: [mario.berrettoni@unicam.it](mailto:mario.berrettoni@unicam.it)

_____________________________________________________________________________________

SUPPLEMENTARY MATERIALS

***S1. Botanical surveys***

The surveys are based on the study of the vegetation, this was carried out through the phytosociological method of the Sigmatist School of Braun Blanquet (1928), which consists in the classical phytosociological study, integrated with the most recent dynamic and ecological reading (**Biondi, 1999; Biondi & Zuccarello, 2000**).

| **ID.** | | | | **1** | **2** | **3** | **4** | **5** | **6** |
| --- | --- | --- | --- | --- | --- | --- | --- | --- | --- |
| Coordinate: Long °E (gg pp ss.dd)  Lat °N (gg pp ss.dd) | | | | 15°29'20.3'' E, 42°06'32.0'' N | 15°29'06.8'' E, 42°06'24.1'' N | 15°29'16.3'' E, 42°07'08.6'' N | 15°29'14.6'' E, 42°07'12.0'' N | 15°29'21.7'' E, 42°06'45.3'' N | 15°28'57.2'' E, 42°06'37.1'' N |
| Name | | | | Grotta del Sale | Cala delle Roselle | Cala tramontana A (internal area) | Cala tramontana B (external area) | Punta del Pigno | Punta di Zio Cesare |
| Exposure | | | | S/SE | SE | W/NW | NW | E/SE | S |
| Slope (°) | | | | 25 | 35 | 25 | 20 | 10 | 15 |
| Coverage (%) | | | | 100 | 100 | 100 | 95 | 100 | 100 |
|  | FormBio | FormCor | Surface (m²) ) | 140 | 140 | 140 | 140 | 140 | 140 |
|  | G bulb | Stenomedit. | *Allium neapolitanum* Cyr. (CFR) |  |  |  |  |  | 3 |
|  | G bulb | Eurimedit. | *Allium vineale* L. (CFR) |  |  | 3 |  |  |  |
|  | G rhiz | Stenomedit. | *Asparagus acutifolius* L. | 5 | 5 | 5 |  | 5 | 10 |
|  | Ch suffr | Endem. | *Centaurea diomedea* Gasp. | 3 |  |  |  | 8 |  |
|  |  |  | *Brachipodium* sp. | 20 | 20 | 5 |  | 30 | 5 |
|  | G rhiz | Europ. | *Carex flacca* Schreber | 5 | 10 |  |  | 3 | 8 |
|  | NP | Stenomedit. | *Cistus monspeliensis* L. | 20 | 3 | 5 |  |  | 3 |
|  | NP | Centro-Europ. | *Coronilla emerus* L. ssp. *emeroides* (Boiss. et Spruner) Hayek |  |  |  |  |  | 5 |
|  | NP | SW-Stenomedit. | *Coronilla valentina* L. |  |  | 3 |  |  |  |
|  | Ch suffr | Eurimedit. | *Crithmum maritimum* L. |  |  |  | 5 |  | 15 |
|  | H caesp | Paleotemp. | *Dactylis glomerata* L. |  |  | 5 |  |  |  |
|  | H bienn | Submedit.-Subatl. | *Daucus gingidium* L. |  |  | 3 | 3 |  |  |
|  | T scap | W-Stenomedit. | *Diplotaxis erucoides* (L.) DC. |  |  | 3 |  |  |  |
|  | H scap | Subatl. | *Diplotaxis tenuifolia* (L.) DC. | 3 | 3 |  |  |  |  |
|  | H caesp | Steno-Medit.-Occid. | Brachypodium retusum (Pers.) P.Beauv. | 30 | 5 |  |  |  |  |
|  | Ch suffr | S-Europ. | *Helichrysum italicum* (Roth) Don |  |  | 3 | 30 |  |  |
|  | P caesp | Eurimedit. | *Juniperus turbinata* Guss. |  |  |  | 30 |  | 8 |
|  | T scap | Eurimedit. | *Lagurus ovatus* L. | 3 | 5 | 3 |  |  |  |
|  | Ch pulv | Endem. | *Limonium diomedeum* Brullo |  |  |  | 5 |  |  |
|  | P lian | Stenomedit. | *Lonicera implexa* Aiton |  |  | 3 |  |  |  |
|  | Ch suffr | Stenomedit. | *Matthiola incana* (L.) R. Br. |  |  |  |  |  | 5 |
|  | H caesp | Stenomedit. | *Melica arrecta* O. Kuntze |  |  |  |  |  | 1 |
|  | H caesp | Paleotemp. | *Melica uniflora* Retz. |  |  |  |  |  | 5 |
|  | G rhiz | Circumbor. | *Milium effusum* L. | 5 | 10 | 5 |  | 8 | 10 |
|  | P caesp | Stenomedit. | *Myrtus communis* L. |  |  |  | 30 |  |  |
|  | P succ | Avv. | *Opuntia ficus-indica* (L.) Miller | 1 |  |  |  |  |  |
|  | T scap | Eurimedit. | *Pallenis spinosa* (L.) Cass. |  | 3 |  |  |  |  |
|  | P caesp | W-Stenomedit. | *Phillyrea angustifolia* L. | 3 | 5 | 3 |  | 3 | 25 |
|  | P scap | Stenomedit. | *Pinus halepensis* Mill. | 50 | 70 | 80 | 10 | 60 | 60 |
|  | P caesp | S-Stenomedit. | *Pistacia lentiscus* L. | 30 | 30 | 30 | 20 | 60 | 30 |
|  | H scap | Stenomedit. | *Reichardia picroides* (L.) Roth |  |  | 3 |  |  |  |
|  | H scap | Europ. | *Reseda lutea* L. |  |  | 3 |  |  |  |
|  | P caesp | Eurimedit. | *Rhamnus alaternus* L. | 8 | 15 | 5 |  | 5 | 15 |
|  | NP | Stenomedit. | *Rosmarinus officinalis* L. | 40 | 5 |  |  | 3 |  |
|  | P lian | Stenomedit. | *Rubia peregrina* L. | 3 | 3 | 5 |  | 5 | 5 |
|  | G rhiz | Eurimedit. | *Ruscus aculeatus* L. |  |  |  |  |  | 3 |
|  | NP | Cosmop. | *Suaeda vera* J.F. Gmel. |  |  | 10 |  |  |  |
|  | H scap | Paleotemp. | *Silene vulgaris* (Moench) Garcke (CFR) |  |  |  | 3 |  |  |
|  | NP | Subtrop. | *Smilax aspera* L. | 5 | 5 | 3 | 5 | 5 | 10 |
|  | T scap | Eurasiat. | *Sonchus asper* (L.) Hill |  | 3 |  |  |  |  |
|  | Ch frut | Stenomedit. | *Stachys major* (L.) Bartolucci & Peruzzi | 3 | 5 | 3 |  | 5 | 3 |
|  | Ch suffr | Stenomedit. | *Teucrium polium* L. | 5 | 3 |  |  |  |  |
|  | G bulb | Stenomedit. | *Urginea maritima* (L.) Baker | 5 | 5 |  |  | 3 | 5 |
|  | NP | S-Medit.-W-Asiat. | *Thymelaea hirsuta* (L.) Endl. |  |  |  | 15 |  |  |

*Table S1.1: Six botanical surveys in San Domino Island. The header of each column, corresponding to a vegetation survey, contains (1) the geographical coordinates of the point, (2) the name of the place, (3) exposure, (4) slope (°), (5) total coverage (%). The columns next to the name of the species show ecological data of the same: FormBio for Biological form (Raunkiaer, 1934) and FormCor for Chorological type (geographic distribution).*

***S2. Flow chamber scheme for soil sampling***


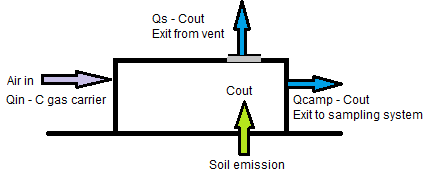


Fig. S2.1-Flow chamber scheme

***S3.Analytical method validation***

The analytical method has been validated for the quantitative analysis of the main analytes sampled with GAC tubes with following solvent extraction and GC-FID analysis.

*Calibration curve*

Two calibration curves were computed using two standards, a-Pinene and Limonene, which, belonging to the same class of compounds, give a similar response factor to GC. Five solutions of known concentration were prepared for each standard, using n-hexane as a solvent: 0.0001 mg/L, 0.0010 mg/L, 0.0100 mg/L, 0.1000 mg/L and 0.5000 mg/L. The solutions were injected four times over four consecutive days. The analytical conditions used with the standards are the same of those used with samples. Below is the calibration curve of α-Pinene – Limonene:

Figure S3.1: Calibration curve of a-pinene

Figure S3.2: Calibration curve of D-limonene

*Linearity*

Linearity was performed after the calibration curves. The goodness of the fit is expressed by the coefficient of determination, R^2^ ≥ 0.99 and signal to noise ratio S/N>10, calculated with GC software from Agilent.

*Sensitivity*

Limit of detection (LOD) and limit of quantification (LOQ) were calculated on the base of standard deviation of the response and slope of calibration curve, using the expressions: 𝐿𝑂𝐷= 3.3𝜎/𝑠𝑙𝑜𝑝𝑒 𝐿𝑂𝑄= 10𝜎/𝑠𝑙𝑜𝑝𝑒, where 𝜎 is the standard deviation of the calibration curve at lowest point (at 0.0001 mg/ml). Table shows the results of sensitivity and values of LOD and LOQ:

| **Compounds** | **RT** | **LOD (mg/ml)** | **LOQ (mg/ml)** | **Sensitivity** |
| --- | --- | --- | --- | --- |
| **α-Pinene** | 12.007 | 0.0001 | 0.0003 | 142029 |
| **Limonene** | 15.716 | 0.0001 | 0.0001 | 144301 |

Table S7.1- Calculated LOD and LOQ for α-Pinene and Limonene

*Precision*

To study the interday precision of the method, four aliquots of the same sample were submitted to the overall developed method in the same weeks and injected in four different days. The Relative Standard Deviation it is expressed in percent and is obtained by the formula RSD% = (standard deviation / mean) x 100.

| Compounds | RSD% | | | | | R^2^ |
| --- | --- | --- | --- | --- | --- | --- |
|  | 0.0001 | 0.0010 | 0.0100 | 0.1000 | 0.5000 |  |
| α-Pinene | 12.5323 | 15.4609 | 12.1151 | 6.7102 | 7.3360 | 0.996 |
| Limonene | 6.7508 | 13.8250 | 12.6954 | 7.6974 | 8.3715 | 0.995 |

Table S3.2 - Calculated RSD% for each value of calibration curve concentration

**QA and QC**: To assess the potential contamination of general Volatile Organic Compounds (VOCs) through cross-contamination and atmospheric deposition, rigorous precautions were taken during the entire laboratory analysis. Nitrile gloves and cotton lab coats were consistently worn throughout the experimental procedures.

For sample collection, Uniphos activated charcoal-sealed tubes were exclusively opened at the moment of sampling. Simultaneously, Carbotrap300 tubes underwent reconditioning through a standard procedure involving helium flow at 250°C, followed by sealing in sample holders and subsequent opening at the time of sampling. Replicating the same sampling sequentially in the field, we observed no discernible differences in the qualitative composition of chromatography, with quantitative variations below 10%.

The entire process of sample collection, transportation, and treatment avoided the use of plastics to mitigate the potential presence of phthalates. Hexane solvent by Merk was distilled before use to eliminate any possible contamination, including other highly volatile components. Blank analyses were conducted to test for the presence of VOCs, and no such compounds were found in the field blanks.

To evaluate accuracy and precision for target compounds Limonene and α-Pinene, we constructed calibration curves and determined analytical parameters, including the Limit of Quantification (LOQ) and Limit of Detection (LOD). Despite the absence of recovery tests, the quantitative information obtained was utilized solely for discerning daily trends, without consideration for absolute quantities. Calculations for LOD and LOQ were based on signal-to-noise ratios (S/N) of 3 and 10, respectively. Concentrations below the Limit of Detection (LOD) were appropriately adjusted by dividing by the square root of two.

***S4.***
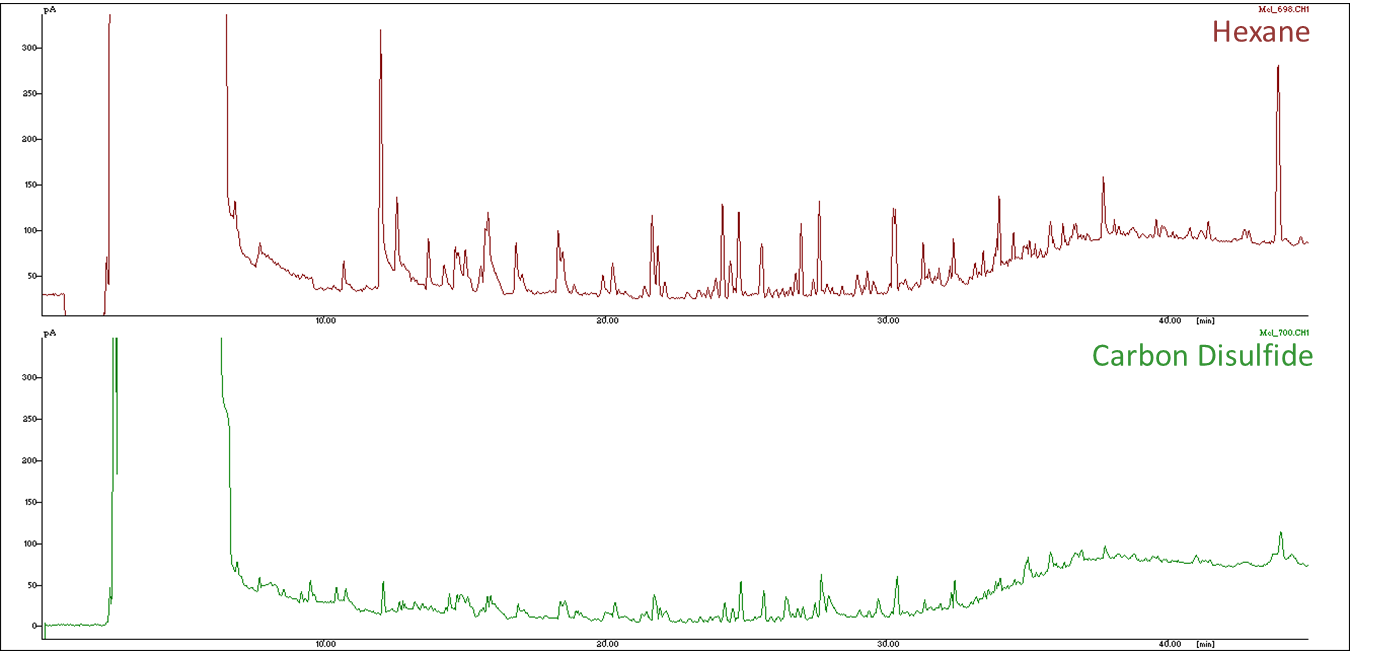
***Optimization of extraction method***

Fig. S4.1- Solvent extraction, comparison between CS_2_ and n-hexane evaluated on the 12h GAC sampled of Rosmarinus officinalis, analyzed with Agilent GC-FID instrument, equipped with an HP-5 column.


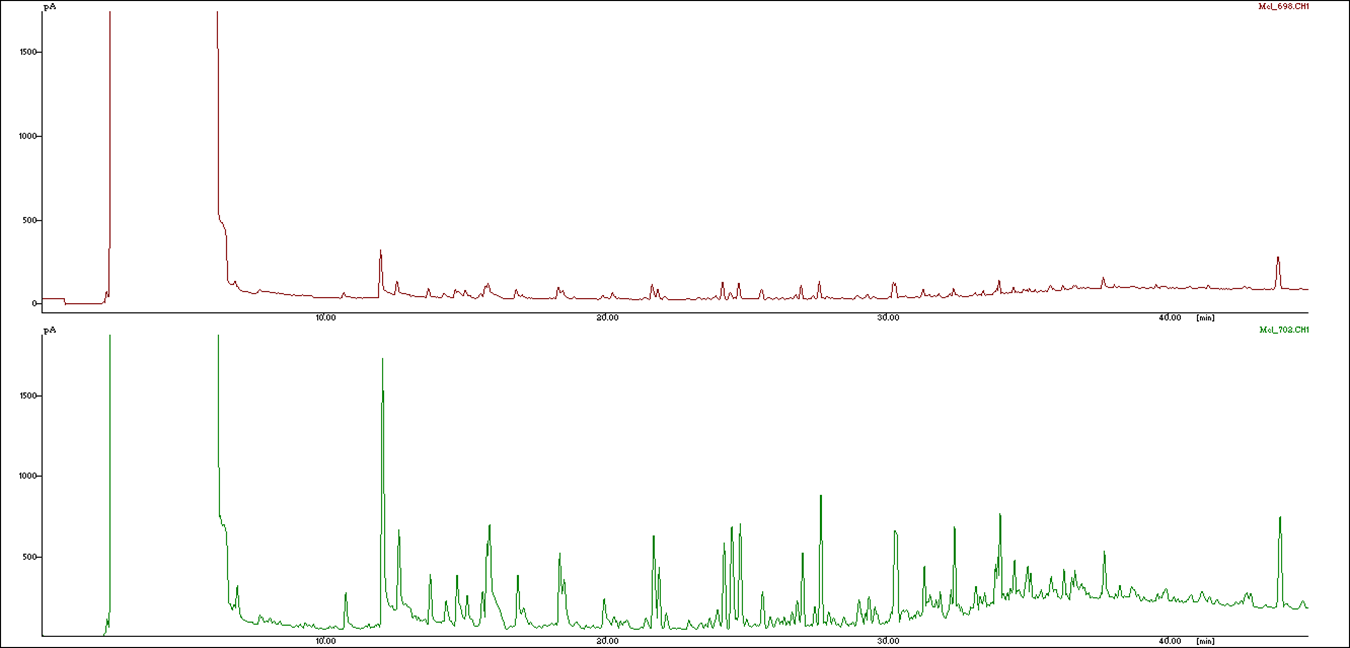


Fig. S4.2-Comparison between simple solvent extraction (red line) and double solvent extraction in ultrasonic bath and concentration (green line) evaluated on the 12h GAC sampling of Rosmarinus officinalis, analyzed with Agilent GC-FID instrument, equipped with an HP-5 column.

The upper chromatogram in figure above shows the result of extraction with solvent, the lower one shows the result using ultrasonic bath and concentration. This procedure increases the quality of the quali/semi-quantitative analysis.

***S5. Terpenes degradation***

Fig. S5.1- Degradation products evaluated after three months from 12h GAC sampling of Rosmarinus officinalis analyzed with Agilent GC-FID instrument, equipped with an HP-5 column.


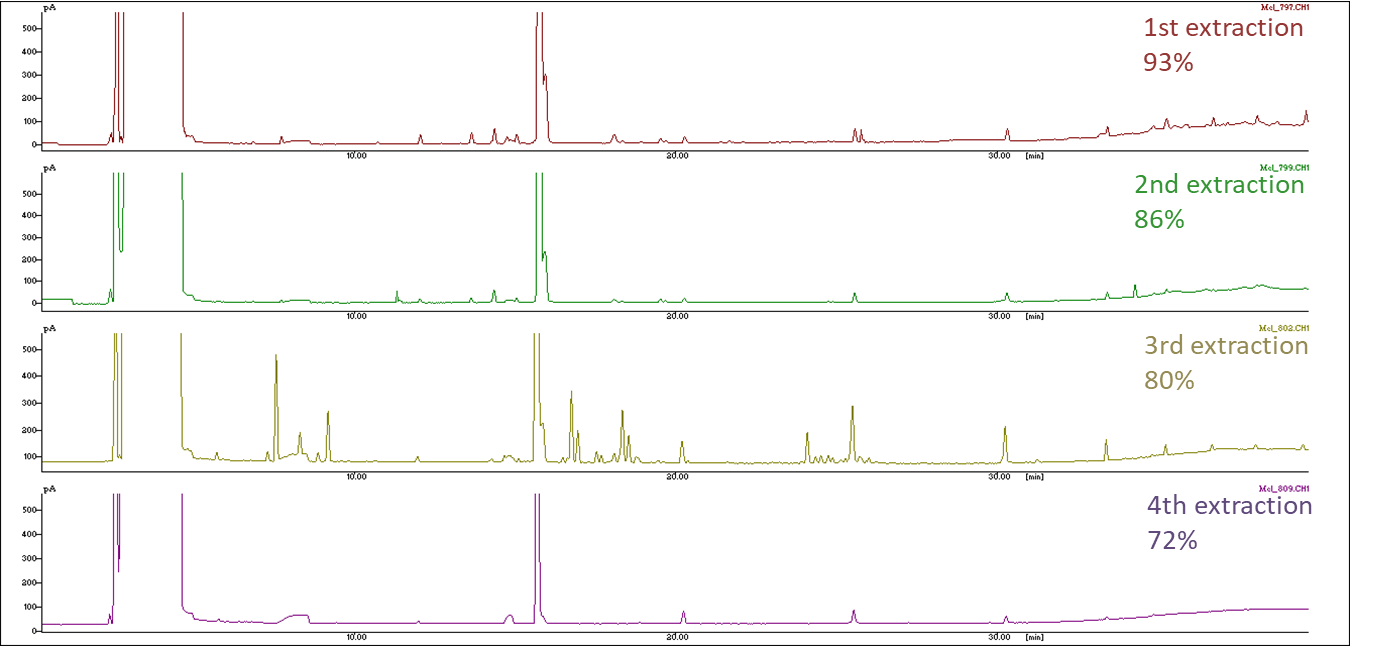
Fig. S5.2- Degradation of adsorbed standard of Limonene on GAC, evaluated with extraction after 4 days, 1 month, 2 months and 3 months storage, analyzed with Agilent GC-FID instrument, equipped with an HP-5 column.

Degradation products

Limonene

Limonene

Limonene

Limonene

**S6.** **Qualitative analysis**

|  |  |  |  |  |  | ID.1 - AIR | ID.1 - SOIL | ID.2 - AIR | | ID.2 - SOIL | | ID.3 - AIR | | ID.3 - SOIL | | ID.4 - AIR | |
| --- | --- | --- | --- | --- | --- | --- | --- | --- | --- | --- | --- | --- | --- | --- | --- | --- | --- |
|  |  |  |  |  |  | GAC | GAC | GAC | TD | GAC | TD | GAC | TD | GAC | TD | GAC | TD |
|  | SE-GC | | | TD-GC | | 70 | 86 | 82 | 55 | 83 | 54 | 78 | 53 | 75 | 52 | 87 | 56 |
| Compound name | KIc | KIa | KIn | KIc | KIo | %  (Area/a.u.) | %  (Area/a.u.) | %  (Area/a.u.) | %  (Area/a.u.) | %  (Area/a.u.) | %  (Area/a.u.) | %  (Area/a.u.) | %  (Area/a.u.) | %  (Area/a.u.) | %  (Area/a.u.) | %  (Area/a.u.) | %  (Area/a.u.) |
| 1,3,8-p-Menthatriene | - | - | - | 1166 | - | - | - | - | - | - | - | - | 0.21  (568684) | - | - | - | - |
| 1-Hexanol,2-ethyl | - | - | - | 1076 | - | - | - | - | - | - | - | - | - | - | 21,05  (23406802) | - | - |
| 1-Octanol | - | - | - | 1117 | - | - | - | - | - | - | 0.90  (1084540) | - | - | - | 1,46  (1625355) | - | - |
| 2-Heptenal | - | - | - | 1014 | - | - | - | - | - | - | 0.55  (662499) | - | - | - | 0,56  (619934) | - | 0,49  (8556392) |
| 3-Carene | 1004 | 1011 | 1011 | 1033 | 1022 | 1.57  (31) | 53.66  (154) | - | 0.79  (341341) | 4.86  (91) | 1.89  (2271599) | - | 3.04  (8039998) | 1.97  (62) | 3,86  (4293039) | 7,82  (285) | 40,92  (713274665) |
| 4-pentenal | - | - | - | 916 | - | - | - | - | - | - | 0.20  (243382) | - | - | - | 0,17  (187736) | - | - |
| allo-Ocimene | - | - | - | 1151 | - | - | - | - | - | - | - | - | 0.09  (237148) | - | - | - | - |
| α-Pinene | 939 | 939 | 937 | 954 | 950 | 15.72  (310) | - | 2.68  (14) | 57.70  (24886768) | 53.09  (995) | 8.79  (10573389) | 64.46  (2053) | 46.40  (12276665) | 58.88  (1854) | 12,75  (14175812) | 10,21  (372) | 0,01  (128897) |
| α−Terpinene | 1027 | 1017 | 1017 | 1043 | 1030 | - | - | - | 0.37  (158320) | - | 0.37  (441320) | - | 0.30  (780614) | - | - | 1,43  (52) | 0,63  (11059187) |
| α−Thujene | 932 | 930 | 927 | 944 | 944 | 3.30  (65) | - | - | 12.69  (5475077) | - | 1.87  (2253183) | - | 0.21  (545300) | - | 0,37  (405878) | 1,54  (56) | 7,63  (132998622) |
| β−cis-Ocimene | - | - | - | 1049 | - | - | - | - | - | - | - | - | 0.11  (295511) | - | - | - | 0,10  (1834820) |
| β−Myrcene | 992 | 990 | 989 | 1002 | 1005 | - | - | - | - | - | 7.17  (8631885) | 10.71  (341) | 36.99  (97860670) | 2.13  (67) | 7,64  (8497588) | - | - |
| β−Phellandrene | - | - | - | 1061 | 1051 | - | - | - | 0.38  (1632830) | - | 0.47  (561913) | - | 0.44  (1167128) | - | 0,36  (401605) | - | 22,69  (395564989) |
| β−Pinene | 981 | 979 | 978 | 1000 | - | 11.97  (236) | - | - | 5.29  (2282558) | 6.83  (128) | - | 3.92  (125) | Trace | 5.97  (188) | - | 6,23  (227) | - |
| Camphene | 954 | 954 | 950 | 976 | 972 | 12.37  (244) | - | - | - | 3.26  (61) | 0.25  (297474) | - | 1.54  (4076202) | 0.79  (25) | 0,22  (249057) | 1,15  (42) | 0,16  (2729851) |
| Camphor | 1136 | 1146 | 1144 | - | - | - | - | - | - | - | - | - | - | - | - | 2,25  (82) | - |
| Decanal | 1209 | 1201 | 1204 | 1255 | - | 4.41  (87) | 15.68  (45) | 16.83  (88) | - | - | 2.38  (2860911) | 1.79  (57) | Trace | 1.37  (43) | 0,22  (249232) | 4,36  (159) | Trace |
| Decanol | - | - | - | 1522 | - | - | - | - | - | - | 0.97  (1164338) | - | - | - | 22,69  (25230251) | - | 1,36  (23717313) |
| D-Limonene | 1033 | 1029 | 1024 | 1053 | 1054 | 5.83  (115) | 13.24  (38) | 42.83  (224) | 3.79  (1632830) | 17.13  (321) | 19.71  (23717313) | 10.99  (350) | 3.40  (9000267) | 16.26  (512) | 1,34  (1489009) | 31,38  (1143) | 4,24  73968540) |
| Dodecanal | - | - | - | 1458 | - | - | - | - | - | - | 2.90  (3491726) | - | - | - | 1,22  (1360423) | - | - |
| endo-Borneol | 1158 | 1169 | 1169 | - | - | - | - | - | - | - | - | - | - | - | - | Trace | - |
| Ethylbenzene | - | - | - | 888 | - | - | - | - | - | - | Trace | - | - | - | Trace | - | 0,02  (322190) |
| Eucalyptol | 1036 | 1031 | 1031 | 1065 | - | 28.30  (558) | - | - | - | - | - | - | 0.24  (638263) | Trace | - | 2,11  (77) | Trace |
| Fenchene | - | - | - | 969 | - | - | - | - | - | - | - | - | Trace | - | - | - | 0,15  (2693566) |
| Fencocamphorone | - | - | - | 1181 | - | - | - | - | - | - | 0.34  (403854) | - | - | - | - | - | - |
| γ−Terpinene | - | - | - | 1076 | - | - | - | - | 1.16  (501302) | - | 22.27  (26805572) | - | 0.22  (576653) | - | - | - | - |
| Heptane, 3-methylene | - | - | - | 794 | - | - | - | - | Trace | - | 9.99  (12016086) | - | 0.08  (217387) | - | 9,71  (10798535) | - | 0,01  (226903) |
| Hexanal | - | - | - | 841 | 840 | - | - | - | - | - | 0.59  (714249) | - | - | - | 0,51  (565747) | - | - |
| Hexanal, 2-ethyl | - | - | - | 994 | - | - | - | - | - | - | Trace | - | - | - | 2,24  (2491449) | - | - |
| Linalool | - | - | - | 1143 | - | - | - | - | - | - | - | - | 0.40  (1051604) | - | - | - | 0,11  (1834820) |
| m-Cymenene | - | - | - | 1117 | - | - | - | - | - | - | - | - | 0.13  (332097) | - | - | - | - |
| Nonanal | 1107 | 1100 | 1104 | 1151 | 1151 | 4.82  (95) | - | 17.21  (90) | - | - | 3.80  (4572351) | 1.41  (45) | - | - | 5,14  (5714579) | 1,18  (43) | - |
| Octanal | - | - | - | 1048 | 1039 | - | - | - | - | - | 1.13  (1357911) | - | - | - | 1,35  (1503704) | - | - |
| o-Cymene | - | - | - | 1017 | - | - | - | - | - | - | - | - | 0.44  (1175654) | - | - | - | - |
| o-Xylene | - | - | - | 927 | - | - | - | - | - | - | 0.61  (735440) | - | Trace | - | 0,57  (635635) | - | - |
| p-Cymene | 1029 | 1024 | 1024 | 1057 | 1059 | 2.74  (54) | - | 10.90  (57) | - | 2.45  (46) | 0.74  (893575) | 2.01  (64) | Trace | 4.70  (148) | Trace | 23,50  (856) | 3,27  (56968321) |
| p-Cymenene | - | - | - | 1112 | - | - | - | - | - | - | - | - | 0.64  (1703224) | - | - | - | - |
| p-Xylene | - | - | - | 896 | 892 | - | - | - | - | - | 0.48  (576393) | - | - | - | 0,45  (494985) | - | - |
| Sabinene | - | - | - | 997 | 991 | - | - | - | 7.46  (3219174) | - | 4.41  (5310392) | - | - | - | 2,24  (2491448) | - | 8,35  (145565263) |
| Santolina triene | - | - | - | 921 | - | - | - | - | - | - | - | - | Trace | - | Trace | Trace | - |
| Terpinolene | - | - | - | 1110 | - | - | - | - | - | - | - | - | 0.41  (1096248) | - | - | - | - |
| Thuja-2,4(10)-diene | - | - | - | 975 | - | - | - | - | - | - | - | - | 0.44  (1157388) | - | - | - | 0,13  (2204778) |
| Toluene | - | - | - | 792 | 791 | - | - | - | 1.67  (719618) | - | Trace | - | Trace | - | Trace | - | 0,14  (2452309) |
| trans-Mentha-2,8-diene | - | - | - | 993 | - | - | - | - | - | - | - | - | 0.17  (462469) | - | - | - | - |
| Tricyclene | 920 | - | 918 | 896 | 892 | - | - | - | - | 1.71  (32) | - | - | Trace | - | - | - | - |
| Undecanal | - | - | - | 1358 | - | - | - | - | - | - | 0.81  (975640) | - | - | - | 0,46  (506251) | - | - |
|  |  | % of identification | | | | 91,02 | 82,58 | 90,44 | 91.30 | 89.33 | 93.58 | 95.29 | 95.90 | 92.06 | 96.59 | 93.16 | 90.41 |
|  |  | n. of components | | | | 10 | 3 | 5 | 11 | 7 | 28 | 7 | 29 | 9 | 27 | 15 | 20 |

Table S6.1: Qualitative result of Activated Carbon (GAC) and Carbotrap300 (TD) analysis. KIc Kovats Index calculates for HP5 column, KIa Kovats Index found in Adams (2017), KIn Kovats index found in NIST (2017), KIo Kovats index from another library.

**S7. PCA of GAC analysis and TD analysis**

Unlike the SPME,for AC and TD the first three components define more than 70% of the system, reaching 99% with the first nine. Take into account the components α-Pinene, 3-Carene and Limonene, describe the samples but only with 78,4%.

- Eigenanalysis of the Covariance Matrix (AC/TD):

PC1 PC2 PC3 PC4 PC5 PC6 PC7 PC8 PC9

Eigenvalue 605,15 434,05 236,89 110,23 97,14 50,48 43,82 26,88 14,31

Proportion 0,372 0,267 0,146 0,068 0,060 0,031 0,027 0,017 0,009

Cumulative 0,372 0,639 0,784 0,852 0,912 0,943 0,970 0,986 0,995

...

- Principal Components (AC/TD):

Variable PC1 PC2 PC3 PC4 PC5 PC6 PC7

a-Pinene -0,807 0,104 -0,378 0,070 -0,266 0,112 -0,017

3-Carene 0,521 0,367 -0,614 0,084 -0,107 0,207 -0,036

Limonene 0,079 -0,816 -0,214 0,158 -0,064 0,014 0,033

Camphor -0,172 0,117 -0,135 0,264 0,890 -0,048 0,007

Decanol 0,036 0,143 0,434 0,302 -0,113 0,120 0,065

Eucalyptolo -0,003 0,024 0,122 -0,717 0,179 0,161 -0,006

p-Cymene 0,057 -0,239 -0,008 -0,050 0,094 -0,383 0,190

...


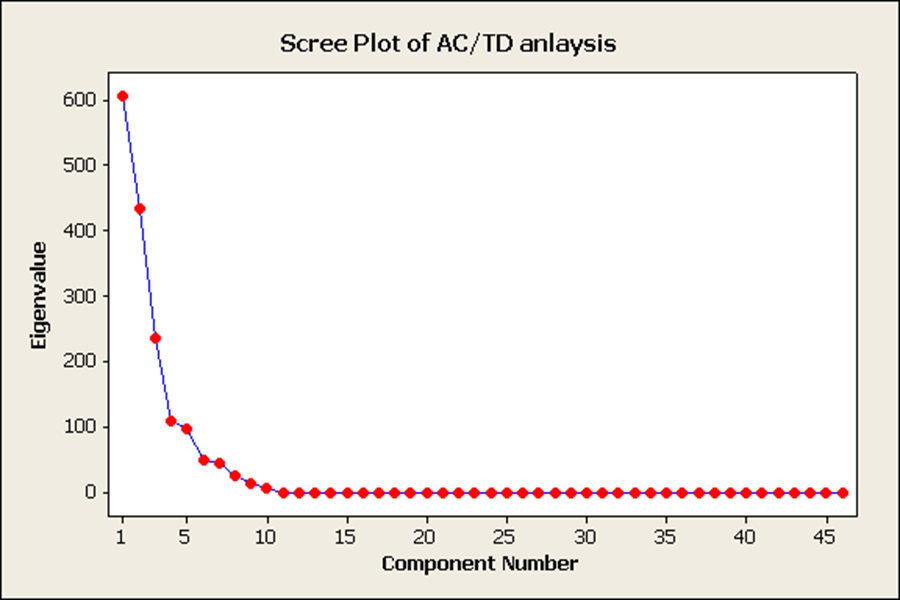


Figure S7.1: Score Plot of GAC/TD

The Score Plot in Figure S7.2, despite the variability of 63.9%, shows the correspondence between the different analyses of TD which is also found in the GAC analysis.


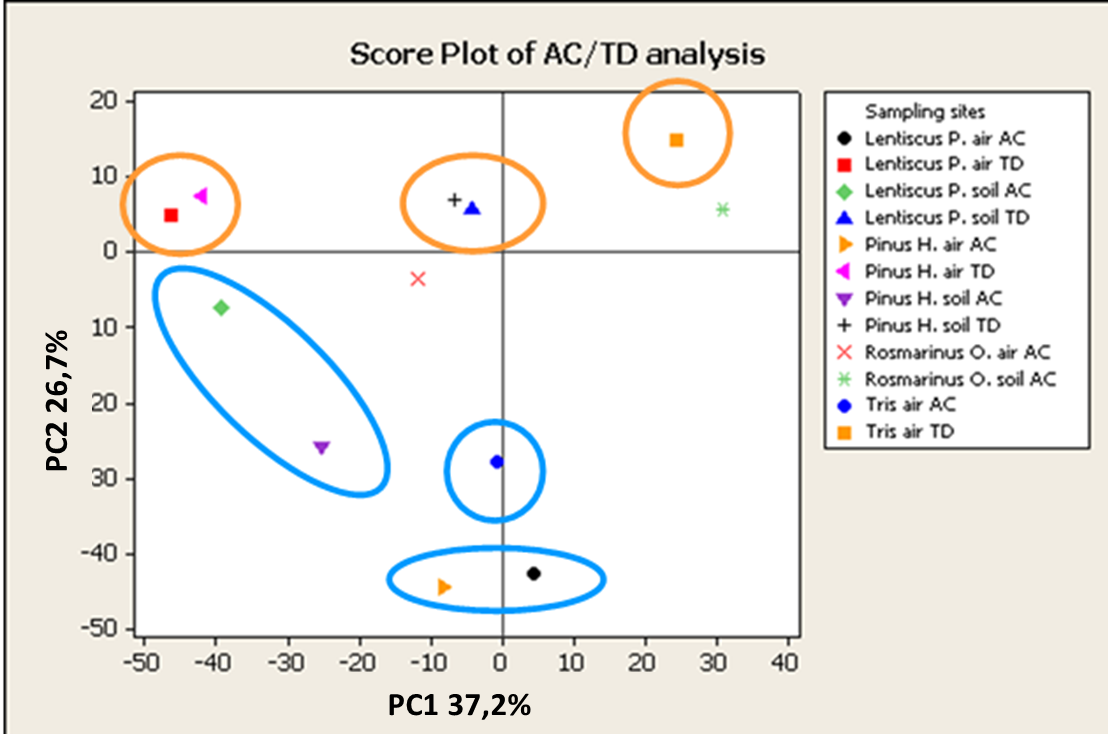


3

2

1

3

2

1

FigureS7.2: Score Plot of GAC/TD analysis

Thermal desorption analyses are grouped with orange circle and divided into subclasses to highlight the matches where "1" highlights the homogeneity in the air analyses of *Pinus H*. and *Pistacia L*. and in the soil in "2"; the group “3” highlights the difference of tris as species and in the sampling site.

As shown in PCA, there is a correspondence between the analysis of the air and soil at the site of abundance of *Pistacia Lentiscus* and *Pinus Halepensis*; this results in BVOCs mixed at the site since the species coexist in the sites (as shown in *Figure S7.3*).


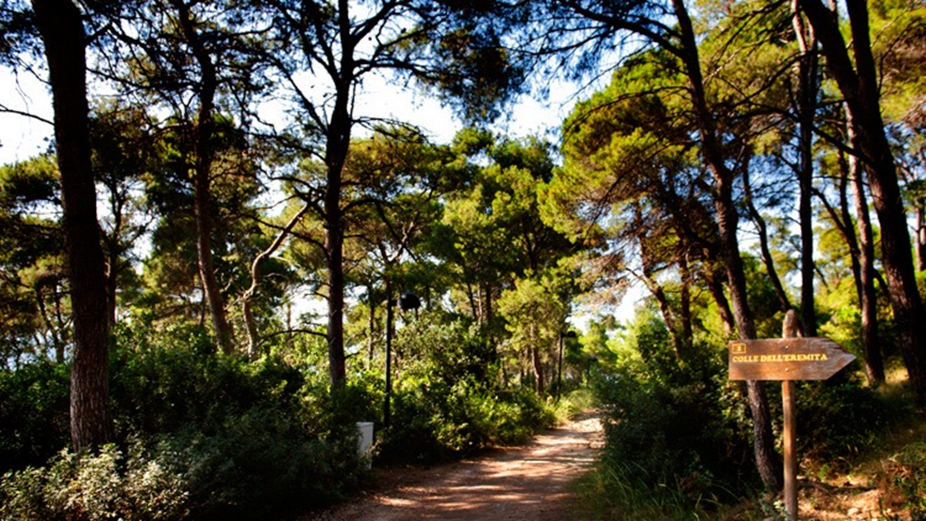


*Pinus Halepensis*

*Pistacia Lentiscus*

Figure S7.3: Perimeter Island of San Domino

With the third component, 78.4% of explained variability is reached. It is also possible in this case to find the same correspondence between *Pinus H*. and *Pistacia L*. of soil and air GAC and TD (green, red, and purple circles). The third component shows correspondence between different types of sampling, tris air GAC and TD (light blue circle).


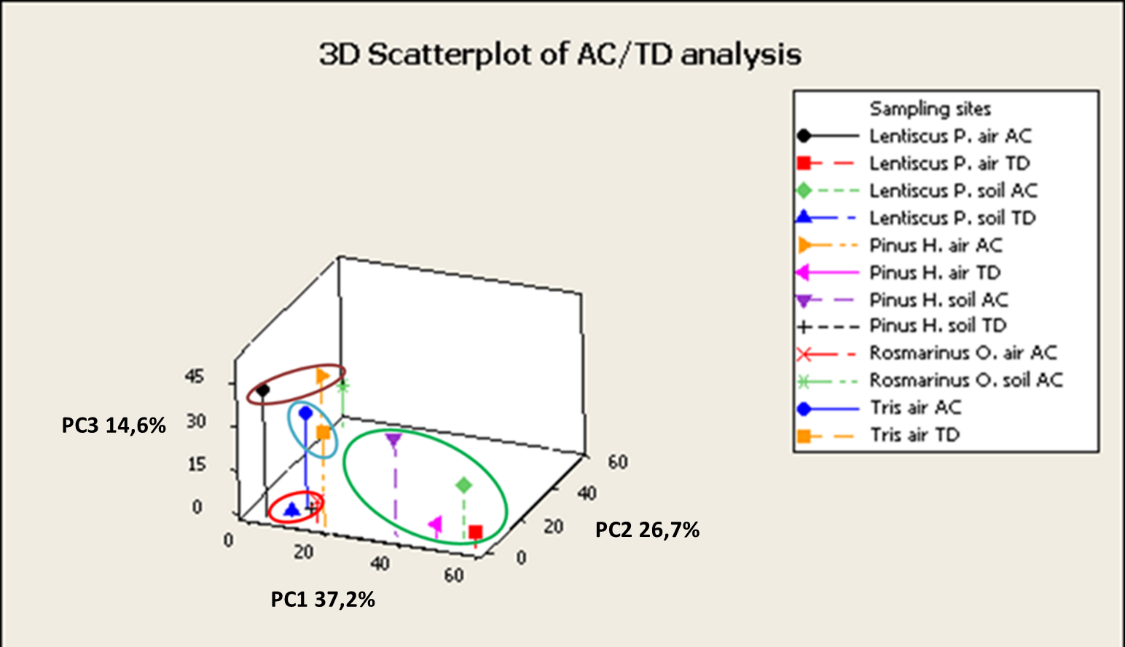


Figure S7.4: 3D Scatter Plot of GAC/TD analysis

The use of two different techniques shows some correspondence in the same analyzes but not always between them, demonstrating a different efficiency and adsorption capacity of adsorbent materials.

**S8. Daily trend with TIGERLT**

Fig. S8.1- Daily trend of total VOCs in air measured with TIGERLT

SXX:
